# Contributions of Jewish Surgeons in the United States

**DOI:** 10.5041/RMMJ.10020

**Published:** 2011-01-31

**Authors:** Seymour I. Schwartz

**Affiliations:** Department of Surgery, University of Rochester Medical Center, 601 Elmwood Avenue, Rochester, New York 14642, USA

**Keywords:** Surgery, Jewish American surgeons, history of surgery

## Abstract

The contributions of Jewish American surgeons in the nineteenth and early twentieth century at a time in which prejudice against ethnic and religious minorities was commonplace in the United States are detailed. The contributions of Jewish American surgeons and the positions they attained subsequent to a change in attitude toward religious minorities in the United States are presented as a comparison.

This historical consideration has as its prime focus on the years that preceded my certification by the American Board of Surgery in 1958. It was a time in which prejudice against ethnic and religious minorities prevented them from being accepted into the surgical and academic mainstream in the United States. The contributions over the past 50 years are included as representatives of achievements of Jewish surgeons during a time in which “Jewish” was erased as a pejorative adjective applied to surgeons.

In the latter half of the nineteenth century and early twentieth century Jewish surgeons in the United States were relegated to hospitals that had been built in urban areas to care for the Jewish population. The first of these hospitals was opened to patients on June 5, 1855 in New York City as the Jews’ Hospital. In 1866, because the hospital was no longer sectarian and so that it was not precluded from receiving state support, the name was changed to the Mount Sinai Hospital.^1^

As would be anticipated, the history of the contributions made by Jewish surgeons in the United States begins with surgeons practicing at that institution. But counter-intuitively, the history begins with a Catholic Hungarian immigrant, Arpad Geza Charles Gerster (Figure 1), who was appointed to the staff of Mount Sinai Hospital in 1880, 3 years after the medical and surgical services were separated at that hospital. From 1882 to 1895, Gerster also held one of the two chairs of surgery at the New York Polyclinic Medical School, the United States’ first post-graduate medical school.

**Figure 1:**
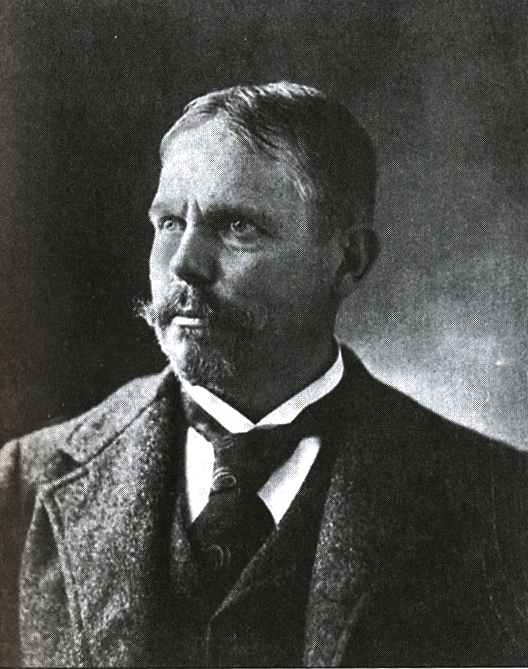
Arpad Geza Charles Gerster.

In 1888, Gerster published *The Rules of Aseptic and Antiseptic Surgery.*^2^ It was the first surgical text to include photographs.^3^ He was also the first to suggest that a surgical procedure might be a factor in the spread of cancer.^4^

Gerster was the first Mount Sinai surgeon to be elected to the American Surgical Association and was honored with the presidency of that organization in 1911. In 1913, his autobiography, *Recollections of a New York Surgeon*,^5^ presented a succinct portrayal of surgery at the time. Gerster’s legacy to Mount Sinai was the cadre of outstanding general surgeons, including Lilienthal, Berg, and Moschcowitz, whom he trained. William J. Mayo remarked that Gerster was “one of the great surgeons of the world”.^6^

In 1887, after the house staff at the Mount Sinai Hospital had been separated into members of either the medical or surgical division, Howard Lilienthal was the first individual to select surgery as his primary interest.^7^ Lilienthal (Figure 2) was born in Albany, New York in 1861 and graduated from Harvard College and Harvard Medical School. He began his tenure at Mount Sinai Hospital as an assistant to Gerster and went on to head a surgical service for 23 years.

**Figure 2:**
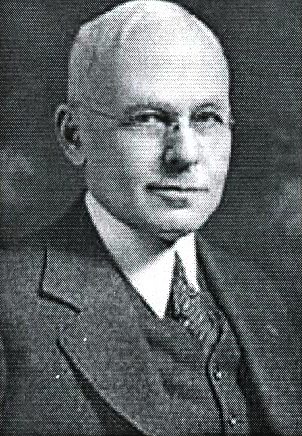
Howard Lilienthal.

Initially, his surgical interests were diverse, and the first of his more than 300 publications was an 1892 paper on the repair of tendons and nerves in the wrist. He was among the first to advocate staged suprapubic prostatectomy and one-stage cholecystectomy for acute cholecystitis. Lilienthal was the first surgeon in the United States to perform a suprapubic prostatectomy,^8^ a colectomy for colitis,^9^ and to administer citrated blood, which had been developed by Richard Lewisohn, to a patient.^10^

Lilienthal was best known as a pioneer in thoracic surgery. In 1910, he reported the first thoracotomy under intratracheal anesthesia, which was administered by Charles Elsberg.^11^ A thoracic service was established at Mount Sinai Hospital in 1914, and that year Lilienthal performed the first successful pulmonary lobectomy in the United States for suppurative disease.^12^ In 1921, he reported extrapleural resection of the thoracic esophagus for carcinoma with the construction of a skin flap to connect the two ends of remaining mediastinal esophagus.^13^ This represented the second report of successful resection of the thoracic esophagus for carcinoma, preceded only by Franz Torek’s case at the German Hospital (now Lenox Hill Hospital in New York City) in which the cervical esophagus and stomach were connected by an external rubber tube to allow oral nutrition.

In 1923, Lilienthal served as the fifth president of the American Association for Thoracic Surgery. In 1925, his primarily single-authored two-volume text-book, *Thoracic Surgery: The Surgical Treatment of Thoracic Disease* was published as the first English compendium on the subject.^14^

Albert A. Berg (Figure 3), after serving as an assistant to Arpad Gerster, was appointed to the staff of Mount Sinai Hospital in 1894 and was chief of the gastrointestinal service of the Department of Surgery from 1915 to 1934. Berg, in response to persuasion by Richard Lewisohn, performed the first subtotal gastric resection for peptic ulcer in the United States. Berg was a strong advocate of the procedure and reported more than 500 cases, in which a recurrence rate of slightly over 1% was compared to a recurrence rate of 34% after gastroenterostomy alone.^15^

**Figure 3:**
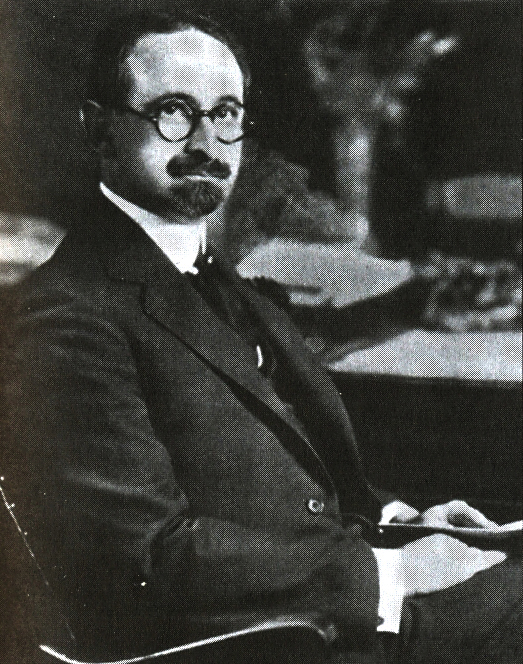
Albert A. Berg.

Berg, who was an indefatigable and extremely facile surgeon, operated on all of the patients included in the landmark report on “Regional ileitis”.^16^ He is perhaps best remembered, along with his brother, for a collection of over forty-thousand books on American and English literature that were gifted to the New York Public Library.

While Berg was the consummate “cutting surgeon”, one of his contemporaries at Mount Sinai Hospital was Richard Lewisohn, a surgeon who, uniquely for the times, focused on research. Lewisohn (Figure 4), who was born and educated in Germany, was chief of the general surgical service from 1928 to 1936. In 1915, he introduced the use of sodium citrate as an anticoagulant, which allowed the effective storage of blood and the subsequent development of blood banks.^17^ Four decades later, he received the Karl Landsteiner Award for that work.

**Figure 4:**
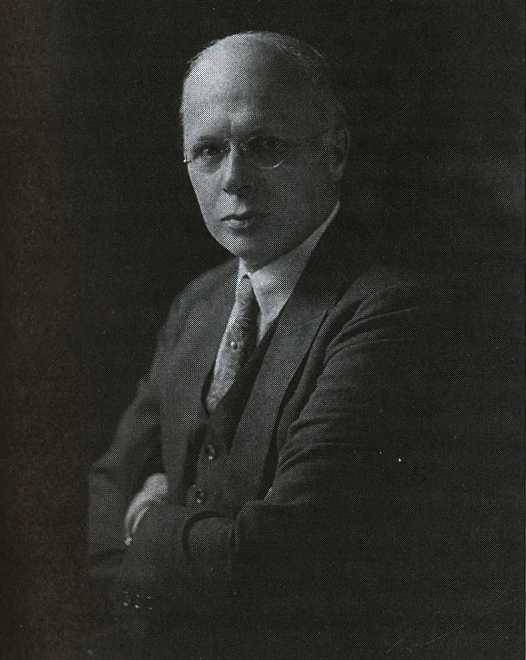
Richard Lewisohn.

After Lewisohn returned from a visit to Han von Haberer in Innsbruck, Austria, where he witnessed a subtotal gastrectomy for a gastric ulcer, Lewisohn convinced Berg to perform the procedure.^18^ Lewisohn reported that subtotal gastrectomy resulted in an acidity, while gastrojejunostomy alone effected no change in gastric acidity, which caused the subsequent development of gastrojejunal ulcers.^19^ In that paper, Lewisohn suggested the possibility of infection as an etiologic factor in the ulcer diathesis, long before the indictment of *Helicobacter pylori*. Lewisohn was also the first to define the significance of folic acid in the biology of cancer and was among the first to use folic acid antagonists clinically.^20^

Alexis V. Moschcowitz (Figure 5), along with Berg and Lewisohn, constituted a surgical triumvirate. He was chief of surgery at Mount Sinai Hospital from 1915 to 1927 and was an active operating surgeon. His name is attached to a technique of repair of femoral hernia and also a repair of rectal prolapse.^21^

**Figure 5:**
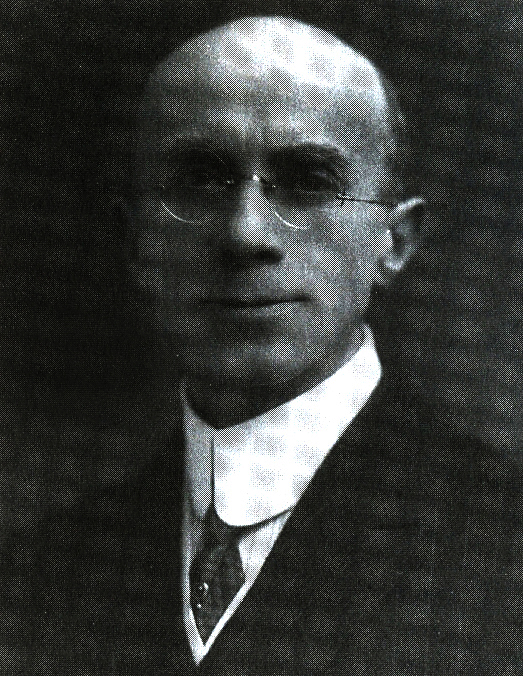
Alexis V. Moschcowitz.

Eponymic status was assigned to two diseases, which were described by surgeons working at Mount Sinai Hospital. Leo Buerger (Figure 6), who eventually focused his activities on urology, in 1908 published a classic description of thrombangiitis obliterans to which his name remains attached.^22^ Leon Ginzburg (Figure 7), who at the time was Berg’s assistant and the hospital’s busiest surgeon, working with Gordon Oppenheimer, a fellow in pathology, defined the lesions of regional enteritis. The disorder was later also dubbed “Crohn’s disease” after the American gastroenterologist Burrill Bernard Crohn who also studied it, but it had been presented to and published by the American Medical Association as “Regional ileitis” in 1932.^23^

**Figure 6:**
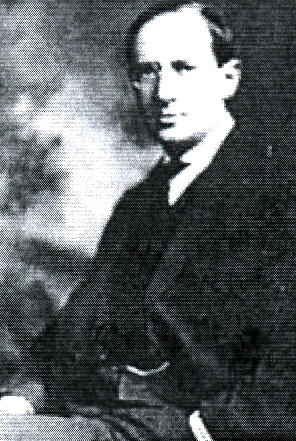
Leo Buerger.

**Figure 7:**
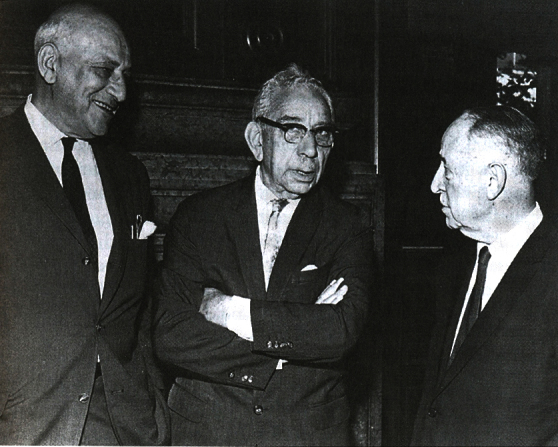
Gordon Oppenhemer, B.B. Crohn, Leon Ginsburg.

Ralph Colp and John H. Garlock continued the tradition of surgical excellence at Mount Sinai Hospital. Garlock was founding member of the American Board of Surgery, the American Board of Plastic Surgery, and the American Board of Thoracic Surgery. In 1952, Mark M. Ravitch (Figure 8), who championed a technique for repair of pectus excavatum and, with David Sabistson, introduced ileoanal anastomosis following total colectomy, became the first full-time Director of Surgery.

**Figure 8:**
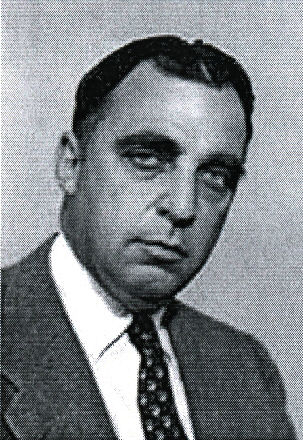
Mark M. Ravitch.

A memorable vignette, which occurred in New York City within the time-span focused upon in this article, stands out. The Jewish surgeon was Rudolph Nissen (Figure 9), a native of Germany, who was the assistant to Ernst Ferdinand Sauerbruch, and was entitled “professor extraordinary” at the Berlin Charité. While in Berlin in 1931, Nissen reported the first successful pneumonectomy, antedating Evarts Graham’s report by 2 years. Unlike Grahams’ one-stage operation, Nissen removed the necrotic lung of a young girl in two stages: first mass ligation of the hilum, followed 14 days later with resection.^24^ The patient was known to be well 16 years later.

**Figure 9:**
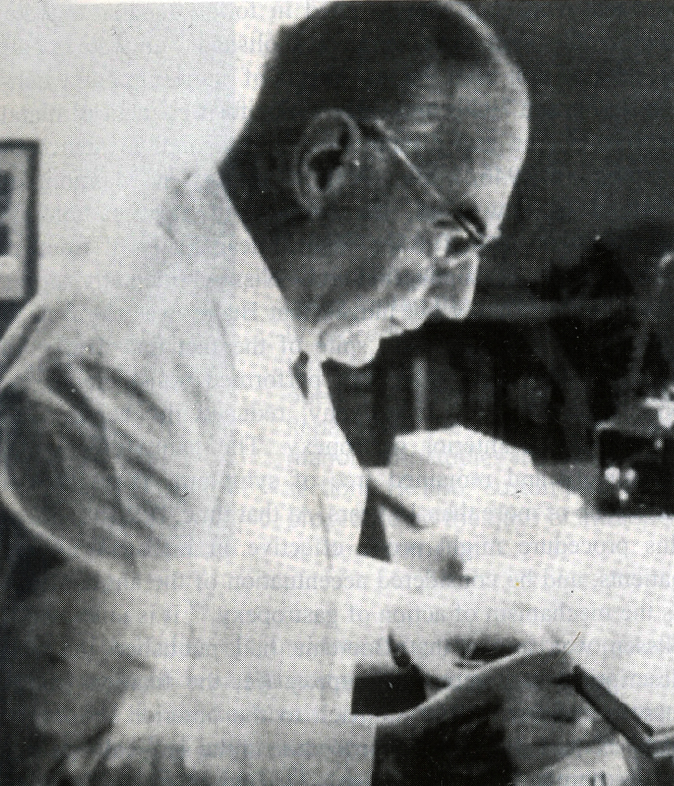
Rudolph Nissen.

Although Nissen did not practice his religion, he appreciated the impending danger associated with the rise to power of the Nazis, and in 1933 assumed the chair of surgery at the University of Istanbul. He emigrated to the United States in 1938 and operated at the Brooklyn Hospital and held the position of Associate Professor of Surgery at Long Island College of Medicine until he left for a professorship at Basel in 1952. Nissen has his name attached to fundoplication for reflux esophagitis, a procedure that he introduced in 1956 with the report of two cases.^25^

In 1948, at the Brooklyn Jewish Hospital, Nissen performed an abdominal exploration for pain on Albert Einstein. Nissen encountered a “grapefruit-sized” aortic aneurysm, which he wrapped anteriorly with cellophane. Einstein lived for 5 more years with minimal discomfort until the aneurysm ruptured and he died.

Surgeons who operated at other hospitals built to satisfy the needs of the Jewish population also made significant contributions. In the realm of vascular surgery, a landmark paper was published based on work performed at the Maimonides Hospital in the Bronx. In 1953, Kenneth Strully, Elliot Hurwitt, and Harry Blankenburg reported a patient with thrombosis of the internal carotid artery, verified angiographically, in whom incomplete removal of the clot was performed. Because they were unable to demonstrate retrograde flow, they ligated and removed the involved segment of the artery. In the article, they concluded that a bypassing procedure or throbo-endarterectomy should be successful if occlusion was localized to the cervical segment of that vessel.^26^

A major contributor to neurosurgery had worked at the same hospital and several others in New York City. Leo M. Davidoff (Figure 10), who had been trained by Harvey Cushing, published the first textbook on pneumoencephalography, which established Davidoff as the father of neuroradiology. In 1955, with the aid of Eugene Spitz and John Holter, Davidoff developed a one-way silicon valve that expedited drainage of the excessive fluid of hydrocephalus.^27^ Davidoff served as chair of the Department of Surgery at the newly established Albert Einstein College of Medicine of Yeshiva University from 1954 to 1958.

**Figure 10:**
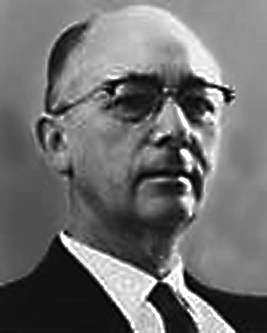
Leo M. Davidoff.

The early roster of the institutions established to satisfy the needs of a Jewish populace included the Beth Israel Hospital in Boston and the Michael Reese Hospital in Chicago. As academic surgery in the United States matured, it identified itself by the formation of a society. The inaugural meeting of the Society of University Surgeons was held in Rochester, New York in 1939, and Samuel J. Stabins (Figure 11), the Jewish chief of surgery at the Genesee Hospital in Rochester, was elected the society’s first president.

**Figure 11:**
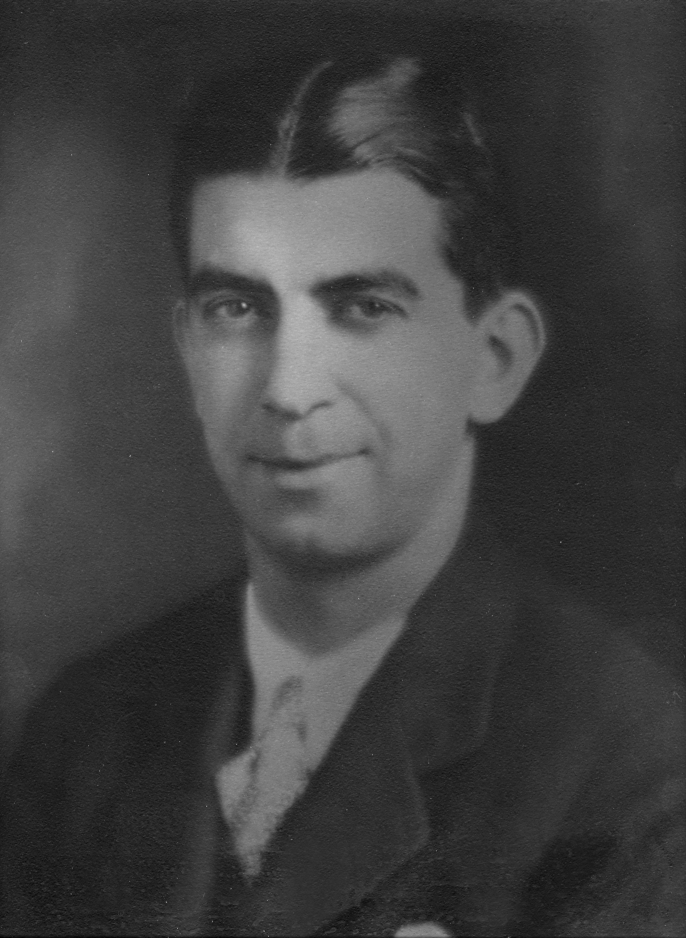
Samuel J. Stabins.

In academia, with the exception of Leo Davidoff at the Albert Einstein College of Medicine, there was but one Jewish chair of surgery at a major university before 1960. In 1956, Leon Goldman (Figure 12), a San Francisco native, was appointed chair of surgery at the University of California. Goldman’s tenure lasted until 1963, and during that period he mentored such notable future surgical leaders as William Blaisdell, Orlo Clark, Alfred deLorimer, John Najarian, Benson Roe, William Silen, and Jack Wylie. Leon Goldman’s daughter, Diane Feinstein, is a United States Senator from California.

**Figure 12:**
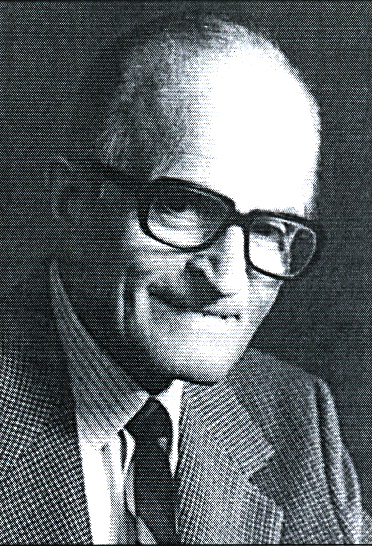
Leon Goldman.

During the past 50 years, significant contributions have continued to be made by American Jewish surgeons. In 1961, Howard A. Frank, who provided the surgical expertise for Paul M. Zoll’s pioneering work on the electrophysiology of the heart, presented the effects of long-term electric stimulation of the heart to the American Surgical Association.^28^ That same year, Adrian Kantrowitz and associates published on the first implantable controllable pacemaker.^29^ Working at the University of Vermont in 1967, Julius H. Jacobson II pioneered the field of microsurgery.^30^ On December 6, 1967, 3 days after Christiaan Neethling Barnard performed the first transplantation of a human heart, Adrian Kantrowitz transplanted the heart from an anencephalic infant into a 2-day old baby at the Maimonides Hospital in Brooklyn. The patient died a few hours after the operation.^31^

In 1994, the American College of Surgeons initiated the Julius H. Jacobson II award for innovation in surgery. Four Jewish surgeons have been among the recipients. Joel D. Cooper received the award in 1996 for his contribution to lung transplantation and lung-volume reduction surgery for emphysema.^32^ In 2004, Judah Folkman, who also had developed the first atrioventricular implantable pacemaker, was honored for his seminal work on angiogenesis.^33^ Four years later, Bernard Fisher was the recipient of the award for his extensive and meticulous study that established breast cancer as a systemic disease and demonstrated that local excision coupled with radiation therapy achieved the same results as radical mastectomy, thereby transforming the surgical approach to the disease.^34^ In 2010, Lazer J. Greenfield became the most recent awardee for his development of an intravenacaval filter to prevent pulmonary emboli while maintaining caval blood-flow.^35^

The ethnic and religious prejudices that restrained surgeons from achieving their full career development and their appropriate recognition for academic and surgical accomplishments are now looked upon as pariahs of the past. A significant number of the major surgical and subspecialty surgical departments are now chaired by Jewish surgeons. The most august of American surgical societies, the American Surgical Association, has had four Jewish presidents. The first served from 1997 to 1998, 117 years after the Association had been formed. The most inclusive American surgical group, the American College of Surgeons, had been presided over by three Jewish surgeons. The first served from 1994 to 1995, 80 years after the College was established. Jewish surgeons have served as members of the Joint Commission on Accreditation of Healthcare Organizations and also have been elected to the Institute of Medicine. Cumulatively, these recognitions provide irrefutable evidence that American surgery has evolved to become a meritocracy, in which ascension to positions of leadership is based solely on one’s contributions and performance.
